# RBR-type E3 ubiquitin ligase RNF144A targets PARP1 for ubiquitin-dependent degradation and regulates PARP inhibitor sensitivity in breast cancer cells

**DOI:** 10.18632/oncotarget.21784

**Published:** 2017-10-10

**Authors:** Ye Zhang, Xiao-Hong Liao, Hong-Yan Xie, Zhi-Min Shao, Da-Qiang Li

**Affiliations:** ^1^ Shanghai Cancer Center and Institutes of Biomedical Sciences, Shanghai Medical College, Fudan University, Shanghai 200032, China; ^2^ Department of Oncology, Shanghai Cancer Center, Shanghai Medical College, Fudan University, Shanghai 200032, China; ^3^ Cancer Institute, Shanghai Cancer Center, Shanghai Medical College, Fudan University, Shanghai 200032, China; ^4^ Department of Breast Surgery, Shanghai Cancer Center, Shanghai Medical College, Fudan University, Shanghai 200032, China; ^5^ Key Laboratory of Breast Cancer in Shanghai, Shanghai Medical College, Fudan University, Shanghai 200032, China

**Keywords:** breast cancer, PARP1, E3 ubiquitin-protein ligase, ubiquitination, proteasomal degradation

## Abstract

Poly(ADP-ribose) polymerase 1 (PARP1), a critical DNA repair protein, is frequently upregulated in breast tumors with a key role in breast cancer progression. Consequently, PARP inhibitors have emerged as promising therapeutics for breast cancers with DNA repair deficiencies. However, relatively little is known about the regulatory mechanism of PARP1 expression and the determinants of PARP inhibitor sensitivity in breast cancer cells. Here, we report that ring finger protein 144A (RNF144A), a RING-between-RING (RBR)-type E3 ubiquitin ligase with an unexplored functional role in human cancers, interacts with PARP1 through its carboxy-terminal region containing the transmembrane domain, and targets PARP1 for ubiquitination and subsequent proteasomal degradation. Moreover, induced expression of RNF144A decreases PARP1 protein levels and renders breast cancer cells resistant to the clinical-grade PARP inhibitor olaparib. Conversely, knockdown of endogenous RNF144A increases PARP1 protein levels and enhances cellular sensitivity to olaparib. Together, these findings define RNF144A as a novel regulator of PARP1 protein abundance and a potential determinant of PARP inhibitor sensitivity in breast cancer cells, which may eventually guide the optimal use of PARP inhibitors in the clinic.

## INTRODUCTION

Breast cancer is one of the most common malignancies and the leading cause of cancer deaths in women worldwide [1]. Accumulating evidence shows that the deregulation of DNA damage response network is intimately implicated in breast cancer pathogenesis and progression [2]. One key regulator of the cellular response to DNA damage is the poly(ADP-ribose) polymerase 1 (PARP1), the most abundant and founding member of the PARP family [3, 4]. PARP1 is activated in response to DNA damage and has essential roles in DNA single-strand break (SSB) repair through the base excision repair (BER) pathway [3–5]. The critical role of PARP1 in DNA repair is manifested by its frequent upregulation in breast cancer cells, which is associated with disease progression and poor survival [6–15]. Rationally, PARP1 represents an attractive target for the development of new anticancer agents against breast cancer.

Indeed, a number of PARP inhibitors have recently been developed for the treatment of breast tumors with homologous recombination (HR) deficiency through a synthetic lethality mechanism, especially those with mutations in the breast cancer susceptibility genes *BRCA1* and *BRCA2*, which encode proteins critical for DNA repair by HR [16–19]. In support of this notion, clinical studies on orally active PARP inhibitor olaparib [20] showed encouraging results in the treatment of triple-negative breast cancer patients carrying tumors with BRCA mutations [18]. Mechanistically, inhibition of PARP1 leads to the conversion of unrepaired SSBs to potentially lethal double-strand DNA breaks (DSBs) during replication [5]. In normal cells, these DSBs are predominantly repaired by the error-free HR repair pathway so that the cells can survive. However, in HR-deficient cells, DSBs are repaired by a more error-prone nonhomologous end-joining (NHEJ) pathway, resulting in chromatid aberrations that usually lead to cell death [5]. Unfortunately, preclinical and clinical evidence has shown that not all patients with BRCA-mutated tumors respond to PARP inhibitors [18], and PARP inhibitors are also effective for breast cancer cells lacking BRCA mutations [21, 22]. Therefore, the identification of novel molecular determinants for cellular sensitivity to PARP inhibitors is critically important for the selection of optimal patients who could potentially benefit from PARP inhibitor therapy. Emerging evidence indicates that, in addition to aberrant expression of HR-related genes [23] and P-glycoprotein drug efflux transporter [19], the protein levels or activities of PARP1 are closely associated with cellular sensitivity to PARP inhibitors [7, 24–28]. Therefore, the assessment of PARP1 expression in tumor samples may improve the selection of breast cancer patients for PARP inhibitor therapy [10]. However, the molecular mechanism underlying the regulation of PARP1 protein levels in breast cancer remains largely unknown.

The ubiquitin-proteasome system is the main protein degradation pathway in the cytosol and nucleus of eukaryotic cells [29]. The ubiquitination of proteins is carried out by an enzymatic cascade consisting of ubiquitin-activating enzymes (E1), ubiquitin conjugating enzymes (E2), and ubiquitin ligases (E3). Of them, E3 ubiquitin ligases play key roles in determining substrate specificity and catalyzing the transfer of ubiquitin from E2 enzymes to the substrate [30, 31]. In humans, there are over 600 E3 ubiquitin ligases, which are classified into three major families, including really interesting new gene (RING), homologous to E6AP carboxyl terminus (HECT), and RING-between-RING (RBR) [31]. In contrast to classical HECT- and RING finger-type E3 ligases, the RBR E3 ligases employ a combined RING/HECT-like mechanism to catalyze the transfer of ubiquitin to a substrate protein [31, 32].

Ring finger protein 144A (RNF144A) is a poorly defined member of the RBR family of E3 ligases [32, 33], which are characterized by the presence of three consecutive structural domains (as known as RBR feature), including an amino-terminal classical RING (RING1), a central in-between-RING (IBR) domain, and a carboxy-terminal RING domain (RING2) [32, 33]. To date, the biological functions and mechanisms of action of RNF144A remain largely unknown. Available evidence suggests that transposable element insertion can induce gene transcription of RNF144A [34], and its single nucleotide polymorphism is associated with adverse effects of antipsychotic medication [35]. More recently, it was reported that RNF144A induces apoptosis in response to DNA damage through targeting DNA-dependent protein kinase (DNA-PK) for ubiquitination and degradation [36], and its ubiquitin ligase activity is regulated by self-association through its transmembrane domain [37]. However, the functional relevance of RNF144A to cancer development and therapeutic response remains undefined.

In this study, we discovered that RNF144A interacts with PARP1 and promotes its degradation through the ubiquitination-proteasome pathway. Moreover, we found that the expression levels of RNF144A in breast cancer cells are associated with cellular sensitivity to PARP inhibitor olaparib. These findings provide novel insights into the regulation of PARP1 protein and may be useful in identifying patients who may be best suited for PARP inhibitor therapy.

## RESULTS

### RNF144A is a novel binding partner of PARP1

**Figure 1:**
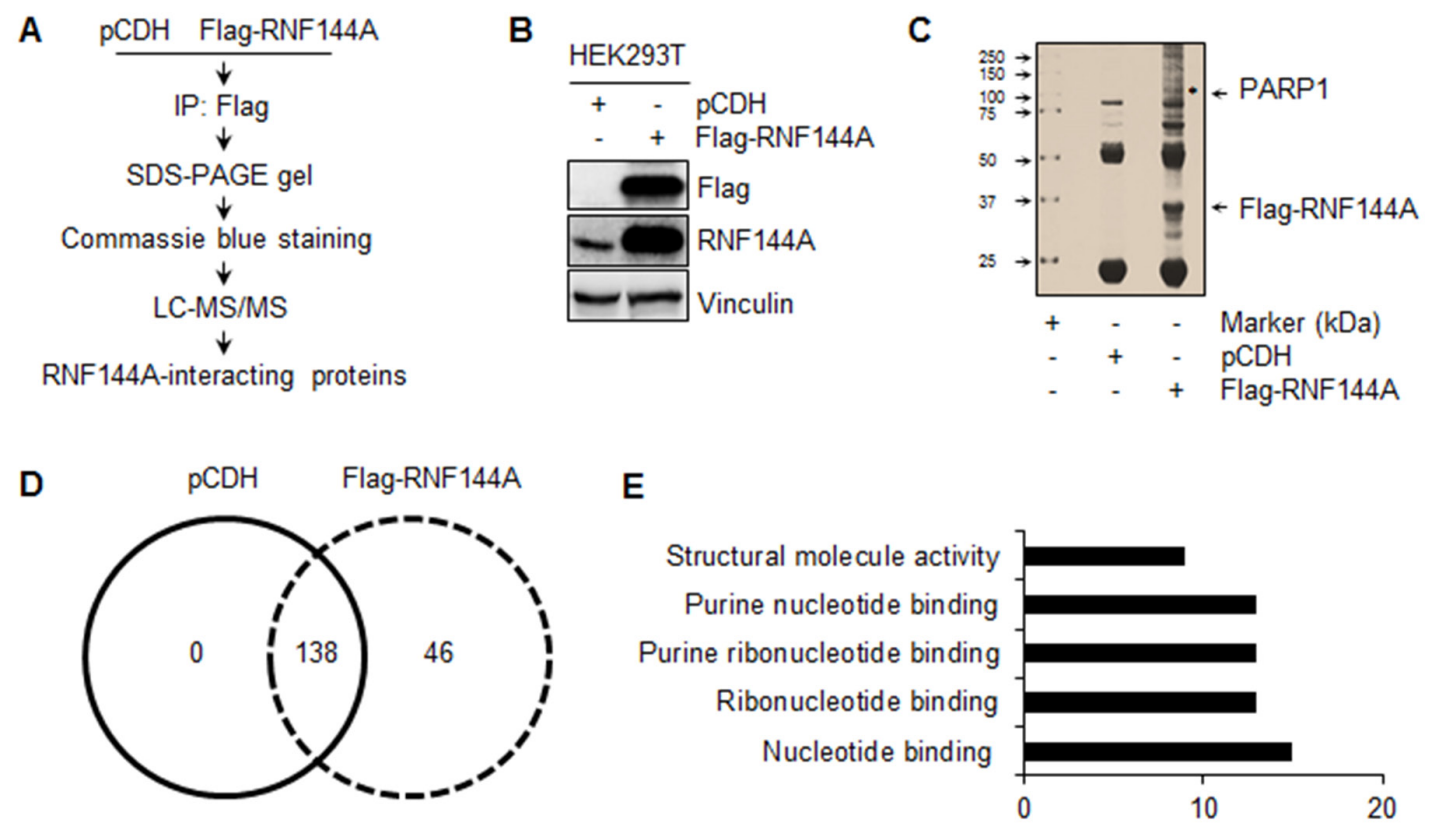
Identification of RNF144A-interacting proteins by LC-MS/MS based proteomics **(A)** Schematic representation of experimental design. **(B)** Total cellular lysates from HEK293T cells stably expressing pCDH and Flag-RNF144A were subjected to immunoblotting analysis with the indicated antibodies. **(C)** HEK293T cells stably expressing pCDH and Flag-RNF144A were subjected to IP analysis with anti-Flag antibody conjugated agarose beads, and the bound proteins were isolated on SDS-PAGE gel and stained using Coomassie brilliant blue. **(D)** LC-MS/MS was used to identify the interacting proteins of Flag-RNF144A. The numbers of the identified proteins in each group are shown. **(E)** Bioinformatic analysis of the biological functions of the proteins that specifically interacted with Flag-RNF144A using DAVID program.

Although RNF144A is widespread in eukaryotes [32, 33], little is known about its biological functions. As E3 ubiquitin ligases play a key role in determining substrate specificity through protein-protein interactions [30, 31], we first identified RNF144A-interacting proteins using a standard immunoprecipitation (IP) coupled with liquid chromatography tandem mass spectrometry (LC-MS/MS) method (Figure 1A). To do this, we generated HEK293T cell lines stably expressing empty vector pCDH and Flag-RNF144A. The expression status of Flag-RNF144A was validated by immunoblotting (Figure 1B). Then, total cellular lysates were subjected to IP analysis with anti-Flag antibody conjugated agarose beads. After being resolved by SDS-PAGE, the precipitated proteins were visualized by Coomassie Blue staining (Figure 1C), followed by LC-MS/MS based proteomic analysis.

On the basis of these analyses, total 46 unique proteins were identified in Flag-RNF144A immunocomplex under highly stringent criteria with a false discovery rate of <1% for peptides and proteins (Figure 1D and Supplementary Table 1). Bioinformatic analysis using the Database for Annotation, Visualization and Integrated Discovery (DAVID) (https://david.ncifcrf.gov/) found that the top 5 biological functions of those 46 proteins are involved in nucleotide binding, ribonucleotide binding, purine ribonucleotide binding, purine nucleotide binding, and structural molecule activity (Figure 1E). Among them, we identified PARP1 as a potential RNF144A-interacting protein that bound to Flag-RNF144A but not empty vector pCDH. As RNF144A is a DNA damage responsive protein [36] and PARP1 is a key player in DNA damage repair [5], we focused on the interaction between RNF144A and PARP1 in this study.

**Figure 2:**
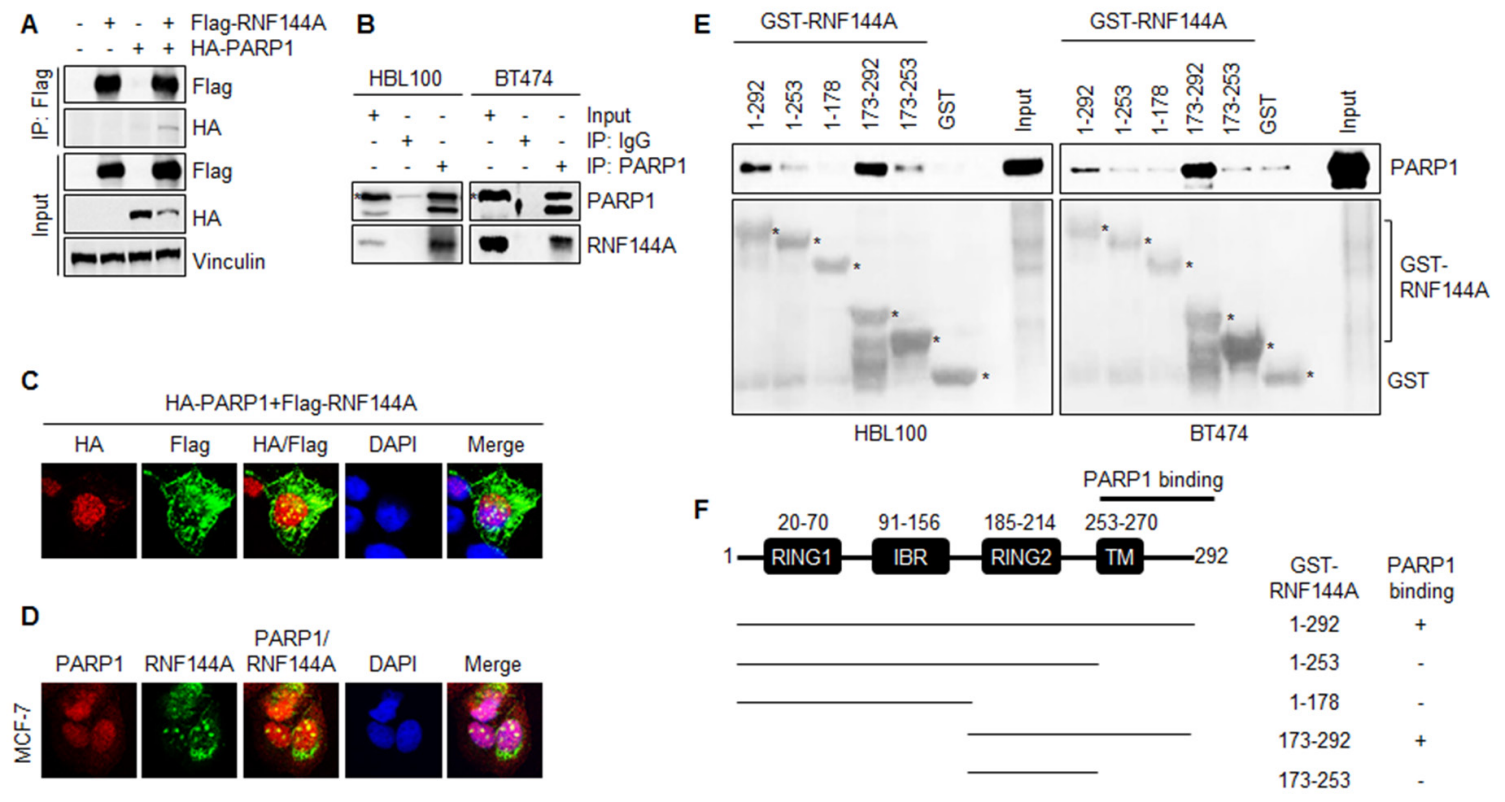
RNF144A interacts with PARP1 through its C-terminal region containing the transmembrane domain **(A)** HEK293T cells were transfected with the indicated expression vectors. After 48 h of transfection, total cellular lysates were subjected to the sequential IP and immunoblotting analysis with indicated antibodies. **(B)** HBL100 and BT474 cells were treated with 10 μM of MG-132 for 4 h and total cellular lysates were subjected to IP and immunoblotting analysis with the indicated antibodies. **(C)** HEK293T cells were cotransfected with Flag-RNF144A and HA-PARP1 and indirect IF staining was performed with the indicated antibodies after 48 h of transfection. Cell nuclei were counterstained with DAPI. **(D)** IF staining of endogenous PARP1 and RNF144A in MCF-7 cells with the indicated antibodies. Cell nuclei were counterstained with DAPI. **(E)** GST or various GST-RNF144A proteins (full length or deletion constructs) were incubated with total cellular lysates of HBL100 and BT474 cells for 4 h, followed by immunoblotting with an anti-PARP1 antibody. **(F)** Schematic representations of RNF144A deletion constructs. The region of RNF144A for PARP1 binding is indicated.

To validate the above proteomic results, HEK293T cells were transfected with Flag-RNF144A and HA-PARP1 alone or in combination and subjected to IP analysis with an anti-Flag antibody. Results showed that exogenously expressed Flag-RNF144A specifically interacted with exogenously expressed HA-PARP1 when co-expressed (Figure 2A). Importantly, we found that endogenous RNF144A interacted with endogenous PARP1 in human breast epithelial HBL100 cells and human breast cancer BT474 cells by IP analysis using an anti-PARP1 antibody (Figure 2B). In addition, there are no commercially available RNF144A antibodies suitable for IP analysis, so we are unable to perform reciprocal co-IP experiments using an anti-RNF144A antibody. Following these observations, we next performed immunofluorescence (IF) staining experiments to examine whether PARP1 and RNF144A could co-localize at both exogenous and endogenous levels. Results showed that PARP1 mainly localizes in the nuclear, while RNF144A is present in both the cytoplasm and the nuclear (Figure 2C and 2D). Consistent with the above proteomic and IP analysis results, exogenously expressed Flag-RNF144A and HA-PARP1 could co-localize in the nuclear in the co-transfected HEK293T cells (Figure 2C). Moreover, RNF144A and PARP1 could co-localize in the nuclear in the MCF-7 cells at the endogenous level (Figure 2D).

Previous studies have documented that RNF144A protein contains four functional domains, including two separated RING-finger domains, an IBR domain, and a transmembrane domain [36, 37]. To map which domain of RNF144A was essential for its interaction with PARP1, GST or various GST-RNF144A deletion constructs were incubated with total cellular lysates from HBL100 and BT474 cells, and resolved on SDS-PAGE, and followed by immunoblotting with an anti-PARP1 antibody. Results showed that PARP1 mainly bound to the C-terminal region of RNF144A (residues 253-292) containing the transmembrane domain (Figure 2E and 2F). Together, these results suggest that RNF144A physically interacts with PARP1.

### RNF144A promotes PARP1 polyubiquitinaiton

**Figure 3:**
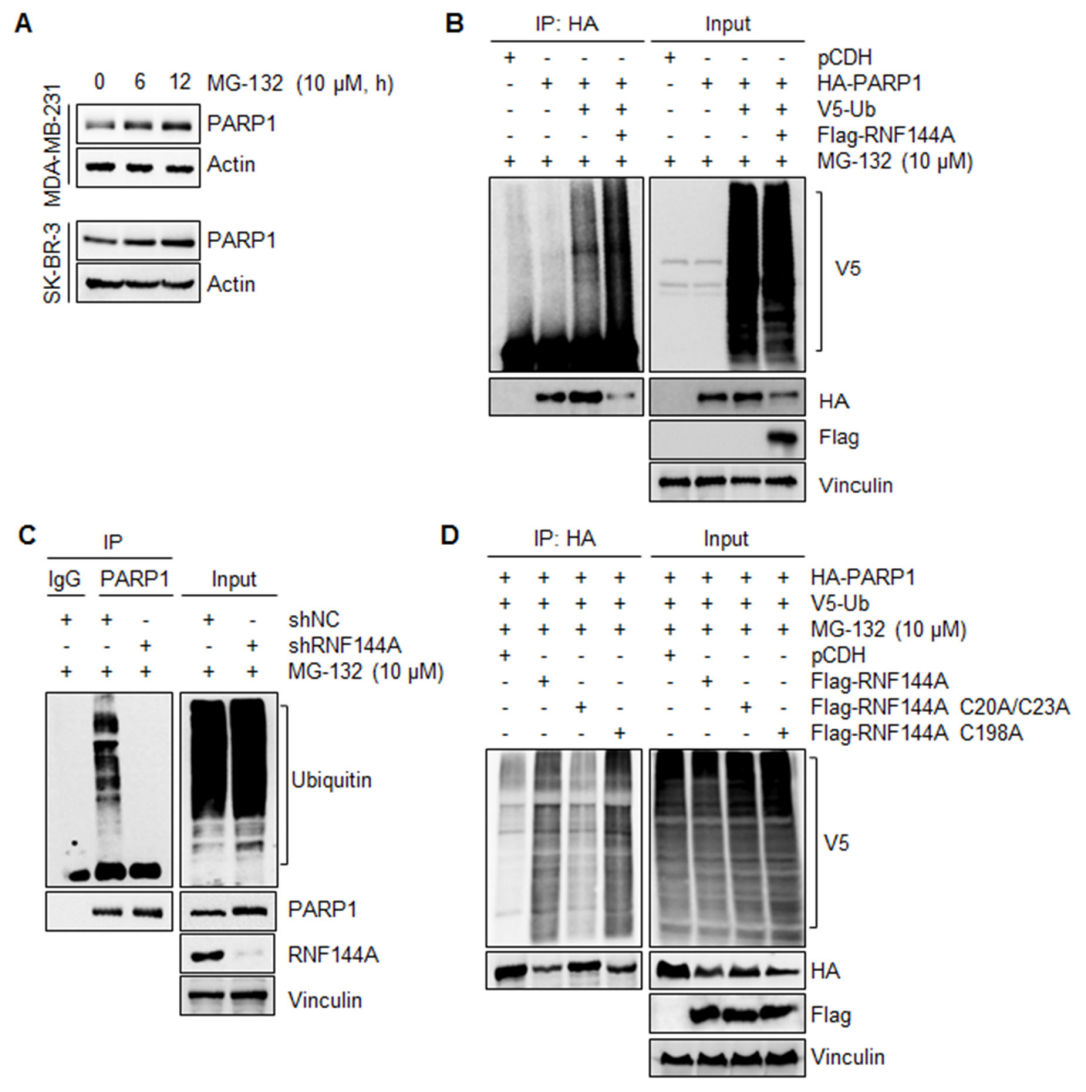
RNF144A promotes PARP1 ubiquitination **(A)** MDA-MB-231 and SK-BR-3 cells were treated with 10 μM of MG-132 for the indicated times and analyzed by immunoblotting with the indicated antibodies. **(B)** HEK293T cells were transfected with the indicated expression vectors. After 48 h of transfection, cells were incubated with 10 μM of MG-132 for 4 h and then subjected to IP and immunoblotting analysis with the indicated antibodies. **(C)** MDA-MB-231 cells expressing shNC and shRNF144A were subjected to IP analysis with an anti-PARP1 antibody or control IgG, followed by immunoblotting with the indicated antibodies. **(D)** HEK293T cells were transfected with the indicated expression vectors. After 48 h of transfection, cells were incubated with 10 μM of MG-132 for 4 h and then subjected to IP and immunoblotting analysis with the indicated antibodies.

As RNF144A is a newly characterized E3 ubiquitin ligase [36, 37], we next investigated whether RNF144A could promote PARP1 ubiquitination. As shown in Figure 3A, treatment of human breast cancer MDA-MB-231 and SK-BR-3 cells with 10 μM of proteasome inhibitor MG-132 resulted in an accumulation of endogenous PARP1 in a time-dependent manner (Figure 3A), indicating that PARP1 undergoes proteasome-dependent degradation. To examine whether RNF144A could promote PARP1 ubiquitinaiton, HEK293T cells were transfected with HA-PARP1, V5-ubiquitin alone or in combination in the absence or presence of Flag-RNF144A. Subsequent IP-Western blot analysis with the indicated antibodies showed that HA-PARP1 was heavily ubiquitinated in the presence of V5-ubiquitin (Figure 3B, left panel, lane 3). Moreover, coexpression of Flag-RNF144A further enhanced HA-PARP1 ubiquitination (Figure 3B, left panel, compare lane 4 with 3). Consistently, knockdown of endogenous RNF144A in human breast cancer MDA-MB-231 cells by specific short hairpin RNAs (shRNAs) targeting human RNF144A (shRNF144A) decreased the ubiquitination levels of endogenous PARP1 (Figure 3C, left panel, compare lane 3 with 2). These results suggest that RNF144A is a novel E3 ubiquitin ligase for PARP1 ubiquitination.

To further substantiate a role of RNF144A in PARP1 ubiquitination, we generated two E3 ligase mutants by substitutions of consensus cysteine (C) to alanine (A) at the residues 20/23 (C20A/C23A) within the RING1 domain (residues 20-70) and 198 (C198A) within the RING2 domain (residues 185-214) [36], and tested their effects on PARP1 ubiquitination *in vivo*. Interestingly, we found that wild-type Flag-RNF144A and Flag-RNF144A C198A, but not Flag-RNF144A C20A/C23A mutant, promoted the appearance of inducible PARP1 ubiquitination (Figure 3D, left panel, compare lanes 2 and 4 with 3), suggesting that the RING1 domain is required for PARP1 ubiquitination.

### RNF144A promotes the proteasomal degradation of PARP1

**Figure 4:**
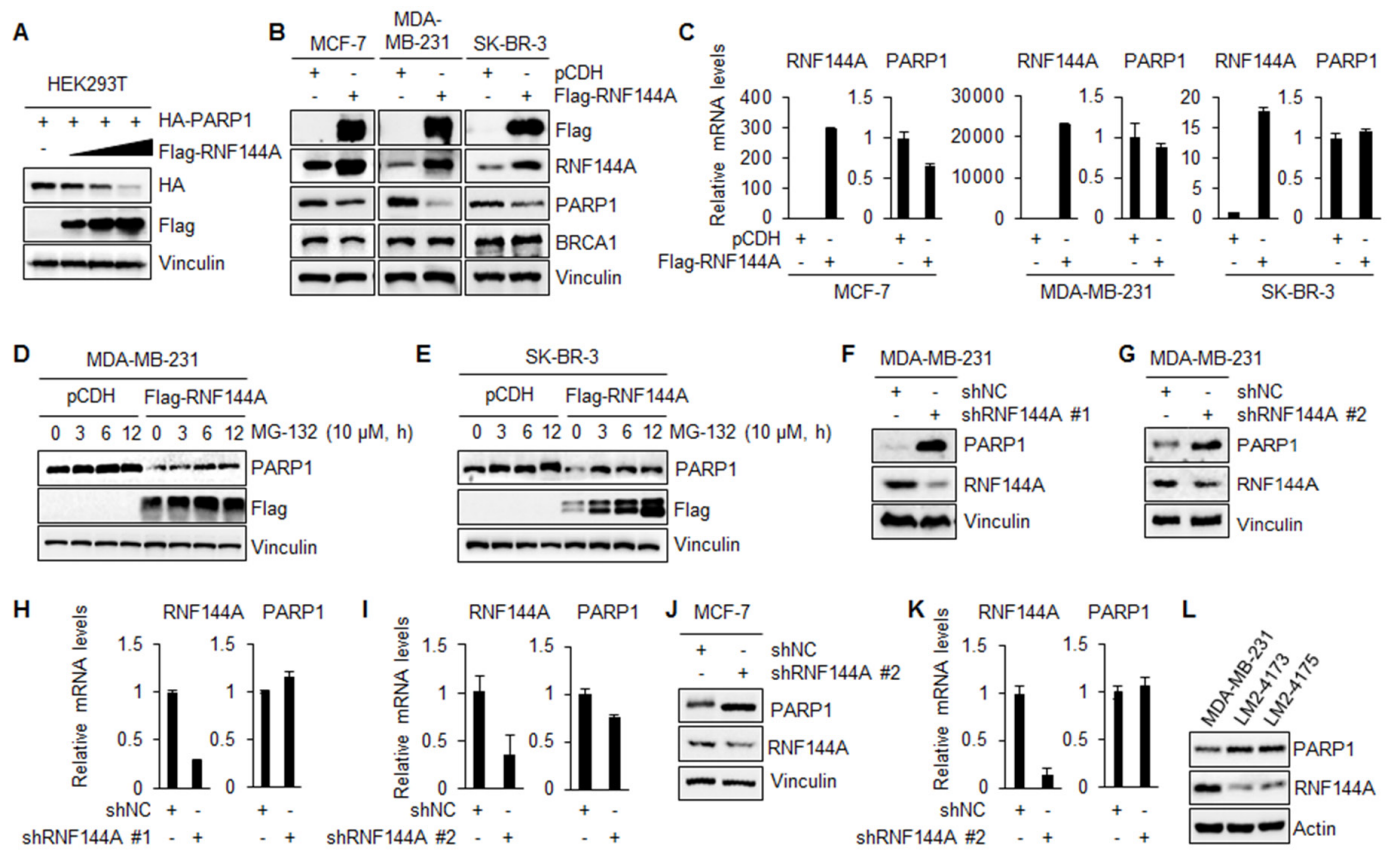
RNF144A promotes the proteasomal degradation of PARP1 **(A)** HEK293T cells were transfected with HA-PARP1 alone or in combination with increasing doses of Flag-RNF144A. After 48 h of transfection, cells were harvested for immunoblotting analysis with the indicated antibodies. **(B-C)** MCF-7, SK-BR-3, and MDA-MB-231 cells stably expressing pCDH and Flag-RNF144A were subjected to immunoblotting analysis with the indicated antibodies (B) or qPCR analysis of the relative mRNA levels of RNF144A and PARP1 (C). **(D-E)** MDA-MB-231 (D) and SK-BR-3 (E) cells stably expressing pCDH and Flag-RNF144A were treated with 10 μM of MG-132 for the indicated time points and analyzed by immunoblotting with the indicated antibodies. **(F-G)** MDA-MB-231 cells stably expressing shNC and shRNF144A #1 (F) and shRNF144A #2 (G) were analyzed by immunoblotting with the indicated antibodies. **(H-I)** Total RNAs were isolated from MDA-MB-231 cells stably expressing shNC and shRNF144A #1 (H) and shRNF144A #2 (I) and analyzed by qPCR. **(J-K)** MCF-7 cells stably expressing shNC and shRNF144A were subjected to immunoblotting (J) and qPCR analysis (K). **(L)** Total cellular lysates of MDA-MB-231, LM2-4173, and LM2-4175 cells were analyzed by immunoblotting with the indicated antibodies.

As ubiquitination of proteins is usually associated with their turnover [29], we next tested whether RNF144A could regulate PARP1 protein abundance. As shown in Figure 4A, coexpression of Flag-RNF144A in HEK293T cells decreased HA-PARP1 protein levels in a dose-dependent manner. Furthermore, stable expression of Flag-RNF144A in human breast cancer MCF-7, MDA-MB-231, and SK-BR-3 cells resulted in a decrease in the protein levels of endogenous PARP1 (Figure 4B). In contrast, induced expression of RNF144A did not significantly affect the protein levels of BRCA1, another key DNA repair protein in breast cancer cells (Figure 4B). Moreover, qPCR analysis showed that the PARP1 mRNA levels were not significantly affected by overexpression of RNF144A in MDA-MB-231 and SK-BR-3 cells (Figure 4C, middle and right panels). In addition, the mRNA levels of PARP1 were slightly down-regulated in RNF144A-overexpressing MCF-7 cells as compared with its pCDH expressing counterparts (Figure 4C, left panel). These results suggest that RNF144A regulates PARP1 expression, at least in part, at the protein level. In support of this notion, MG-132 treatment partially restored RNF144A-mediated downregulation of PARP1 in both MDA-MB-231 and SK-BR-3 cells in a time-dependent manner (Figure 4D and 4E), suggesting a mechanistic role of the ubiquitin-dependent proteasome pathway in RNF144A-mediated downregulation of PARP1.

To further confirm that RNF144A regulates PARP1 protein levels, two different RNF144A shRNAs targeting different sequences within RNF144A gene were transfected into MDA-MB-231 cells. Immunoblotting and qPCR analysis demonstrated that knockdown of endogenous RNF144A resulted in an increase in the protein levels of endogenous PARP1 (Figure 4F and 4G). In contrast, there was no significant difference in the relative mRNA levels of PARP1 between shNC- and shRNF144A-transfected cells (Figure 4H and 4I). In addition, the effects of shRNA-mediated knockdown of endogenous RNF144A on the protein and mRNA levels of PARP1 were also observed in human breast cancer MCF-7 cells (Figures 4J and 4K, respectively). To demonstrate the physiological significance of RNF144A regulation of PARP1, we next analyzed the expression levels of endogenous RNF144A and PARP1 proteins in an isogenic breast cancer progression model system, including parental MDA-MB-231 and its two highly metastatic variants LM2-4173 and LM2-4175 [38]. Results showed that the protein levels of PARP1 were gradually up-regulated, while the levels of RNF144A protein were down-regulated from parental MDA-MB-231 to highly metastatic LM2-4175 cell line (Figure 4L). Collectively, these results suggest that RNF144A is a negative regulator of PARP1 expression.

### RNF144A regulates breast cancer cellular sensitivity to PARP inhibitor olaparib

**Figure 5:**
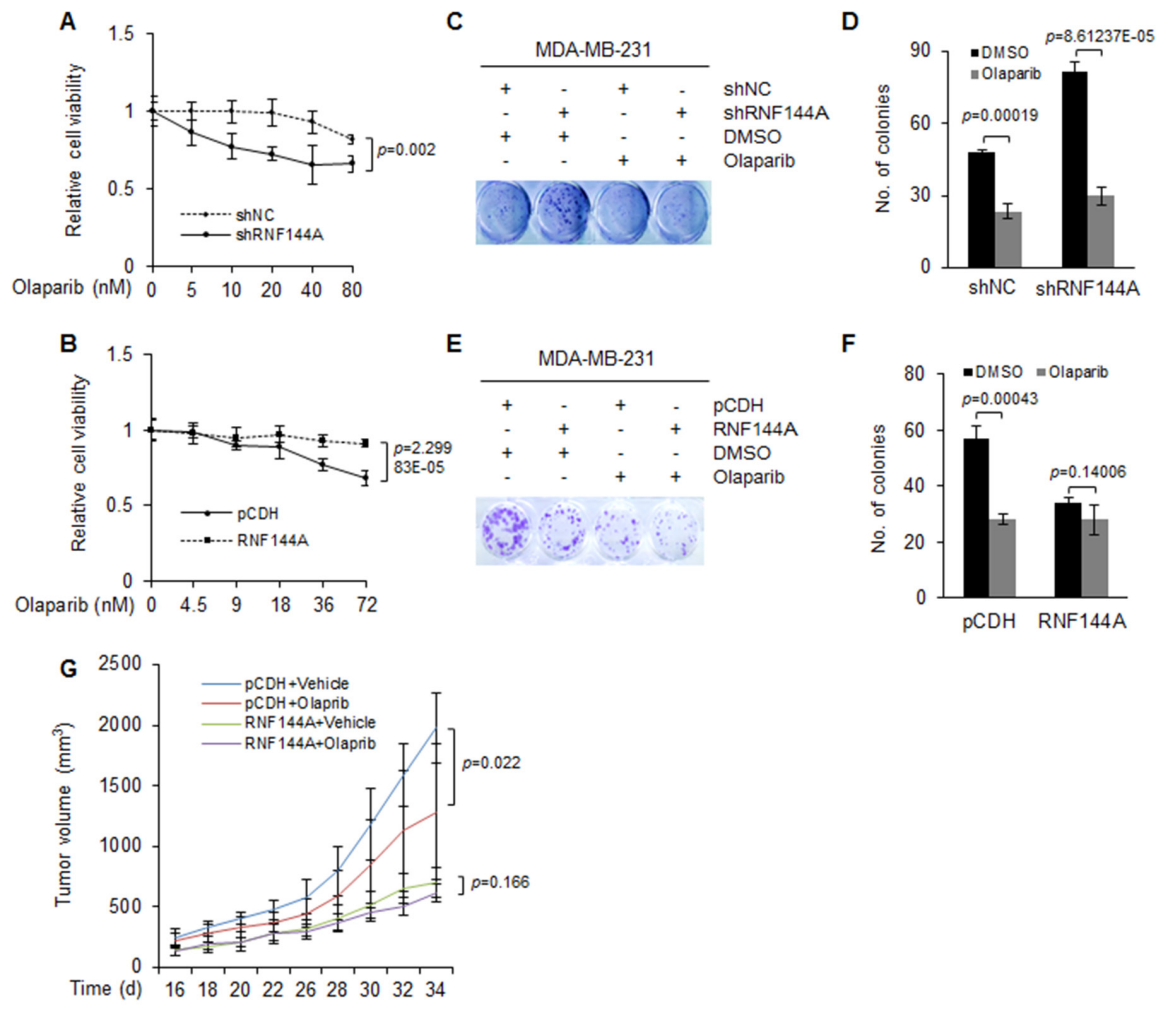
The expression levels of RNF144A are associated with cellular sensitivity to PARP inhibitor olaparib *in vitro* and *in vivo* **(A)** MDA-MB-231 cells stably expressing shNC and shRNF144A were treated with DMSO or olaparib at the indicated doses for 48 h. Cell viability was analyzed using CCK-8 kit. Analysis of cell viability at each drug dose was expressed as a percentage of cells remaining compared with DMSO treatment group. **(B)** MDA-MB-231 cells stably expressing pCDH and Flag-RNF144A were treated with DMSO or olaparib at the indicated doses for 48 h. Cell viability was analyzed using CCK-8 kit. Analysis of cell viability at each drug dose was expressed as a percentage of cells remaining compared with DMSO treatment group. **(C-D)** MDA-MB-231 cells stably expressing shNC and shRNF144A were treated with DMSO or 1 μM of olaparib for 7 days. The colonies were fixed and stained with 0.5% crystal violet (C). Quantitative results are shown in D. **(E-F)** MDA-MB-231 cells stably expressing pCDH and Flag-RNF144A were treated with DMSO or 2.5 μM of olaparib for 7 days. The colonies were fixed and stained with 0.5% crystal violet (E). Quantitative results are shown in F. **(G)** MDA-MB-231 cells stably expressing pCDH and Flag-RNF144A (1 × 10^7^ cells) were injected subcutaneously into the mammary fat pads of 6-8 week old female BALB/c nude mice (n=6). When the tumor volume reached to about 200 mm^3^, mice were administrated via intraperitoneal injection with olaparib (50 mg/kg) or vehicle alone for the indicated times. Tumor volume is shown.

As the protein levels or activities of PARP1 are closely associated with cellular sensitivity to PARP inhibitors [7, 24–28] and RNF144A regulates PARP1 protein abundance (Figures 3 and 4), we next examined whether RNF144A could influence the sensitivity of breast cancer cells to PARP inhibitor olaparib [20], an FDA-approved targeted therapy for human cancers. Triple-negative breast cancer (TNBC) is a very aggressive disease and currently lacks effective treatment options. Emerging evidence shows that PARP inhibitors may have significant anti-tumor effects in this subtype of breast cancer [39]. Therefore, we next examined cell proliferation and tumor growth of RNF144A-depleted or overexpressing MDA-MB-231 TNBC cells in the absence or the presence of olaparib. Cell viability assays showed that shRNA-mediated knockdown of endogenous RNF144A rendered MDA-MB-231 cells more sensitive to olaparib (Figure 5A). In contrast, RNF144A-overexpressing MDA-MB-231 cells were more resistant to olaparib than empty vector expressing control cells (Figure 5B). Colony formation assays demonstrated that knockdown of RNF144A increased the clone number in DMSO-treated cells. Moreover, RNF144A-depleted cells were more sensitive to olaparib than shNC-expressing cells (Figure 5C and 5D). In addition, induced expression of RNF144A decreased the clone number in DMSO-treated cells, and olaparib treatment suppressed cell growth in empty vector pCDH expressing cells (Figure 5E and 5F). However, RNF144A overexpressing cells did not significantly respond to olaparib (Figure 5E and 5F), indicating that expression of RNF144A decreases cellular sensitivity to olaparib *in vitro*.

To evaluate whether RNF144A affects breast cancer cellular sensitivity to olaparib *in vivo*, MDA-MB-231 cells stably expressing pCDH and Flag-RNF144A were subcutaneously injected into 6-8 week old female BALB/c nude mice. When average tumor volumes reached 200 mm^3^, mice were administrated with 50 mg/kg olaparib [40] or vehicle alone (n=6). Each animal received one daily drug administration for five consecutive days, followed by 2 days of no treatment. Consistent with *in vitro* results, induced expression of RNF144A significantly decreased tumor growth in vehicle-treated mice, and treatment of olaparib suppressed tumor growth in mice injected with empty vector expressing MDA-MB-231 cells (Figure 5G). However, mice bearing RNF144A-overexpressing tumors did not significantly respond to olaparib treatment (Figure 5G). This result indicates that tumors expressing high levels of RNF144A are resistant to olaparib.

## DISCUSSION

Multiple lines of evidence have documented that PARP1 is upregualted in breast cancer [6–15]. Consequently, PARP1 enables to compensate the impaired DNA repair and the tumor cells can survive and progress despite of their presence of DNA damage [5, 16]. In addition, PARP1 also plays key roles in gene transcription, which also contributes to cancer development and progression [41]. Despite its basic biological and clinical importance, the underlying mechanisms for the overexpression of PARP1 in breast cancer remain poorly defined. Emerging evidence shows that PARP1 undergoes post-translational modification by ubiquitination and proteasome-dependent degradation [42, 43]. To date, two RING-type E3 ubiquitin-protein ligases for PARP1 ubiquitination under the conditions of heat shock and mitotic stress have been identified, termed checkpoint with forkhead and ring finger domains (CHFR) [42] and ring finger protein 4 (RNF4) [43].

In the present study, using LC-MS/MS based proteomics and Co-IP assays, we identified PARP1 is a novel binding partner of RNF144A (Figures 1 and 2). GST pull-down further demonstrated that the interaction of RNF144A with PARP1 is mediated through its C-terminal region containing the transmembrane domain. Intriguingly, the transmembrane domain seems to play an important role in regulation of RNF144A E3 ligase activity and physiological function [36, 37]. A series of biochemical assays further demonstrated that RNF144A functions as an E3 protein ligase for PARP1 ubiquitination and subsequent proteasomal degradation (Figures 3 and 4). Our recent studies demonstrated that RNF144A is downregulated in breast cancer (manuscript in preparation), which may provide a molecular basis of why PARP1 is upregulated in breast cancer at the protein level. In addition, although a recent study documented that both RING1 and RING2 domains within RNF144A are required for DNA-PK ubiquitination by RNF144A [36], we showed that RING1, but not RING2, is essential for RNF144A-mediated PARP1 ubiquitination (Figure 3D). RING1 has a classical RING fold, which is typically used for E2-E3 interactions [32]. In addition, the IBR-RING2 domain of Parkin, another RBR-type E3 ligase, can mediate the formation of ubiquitin linkages in the absence of RING1 [44]. Thus, although an intact RBR domain is necessary for efficient E3-ligase functioning, RING1 and RING2 are differentially involved in the RBR-type E3 ubiquitin ligase-mediated ubiquitination of proteins in a substrate dependent manner.

Another important issue in this field is the identification of the molecular determinants for cellular sensitivity to PARP inhibitors, which is critical for selecting patients who could potentially benefit from PARP inhibitor therapy. Previous studies have shown that olaparib has a considerable effect in HR repair-deficient breast cancers [17]. In addition to HR repair defects, emerging evidence highlights that the protein levels or activities of PARP1 itself are closely associated with cellular sensitivity to PARP inhibitors [7, 24-28, 45]. Consistently, PARP1 is hyperactivated in HR-defective cells, which is correlated with an increased sensitivity to PARP inhibitors [26]. Clinical trial data also showed a dose-dependent clinical response to PARP inhibitor therapy [46], suggesting that it may be worthwhile to consider the amount of PARP expression in tumor cells. Consistently, BRCA mutated TNBC cell lines that are sensitive to PARP inhibition express high levels of PARP1 [47], and cell lines that are sensitive to olaparib are enriched in PARP1 amplification in addition to other genetic alternations [25]. Therefore, future clinical trials involving PARP inhibitors should take into account not only constitutional genetic background but also PARP1 protein expression in breast cancer cells [10, 45].

**Figure 6:**
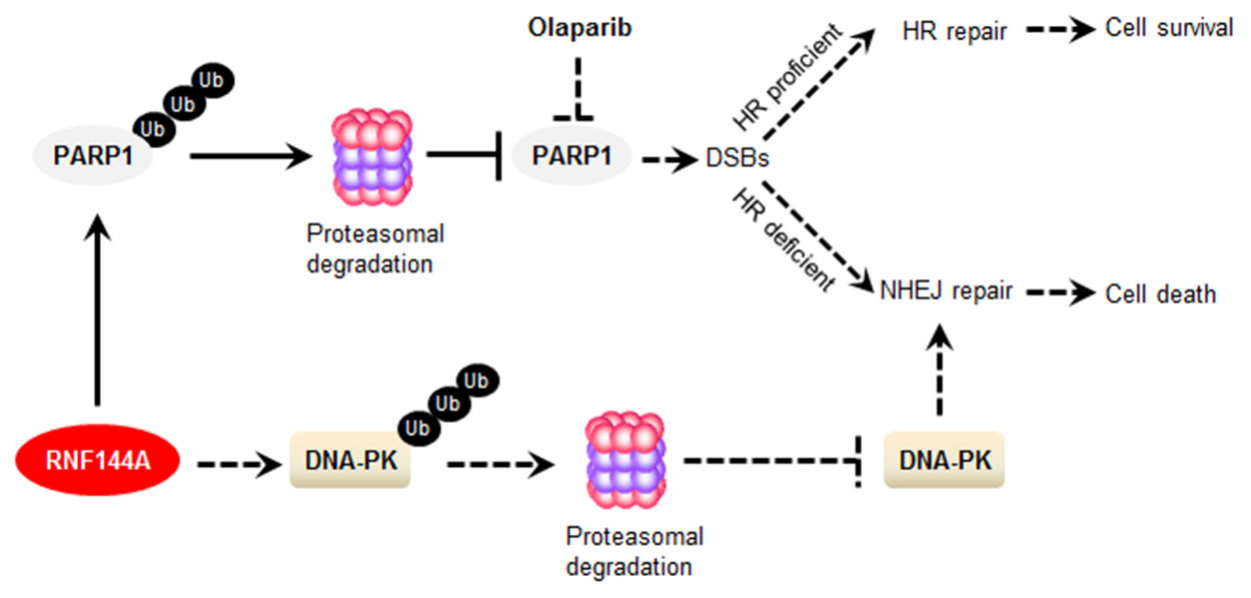
The proposed working model PARP1 is essential for DNA repair through the BER pathway. PARP inhibition by olaparib results in the formation of DSBs. In HR-proficient cells, these DSBs would be repaired by the error-free HR repair pathway, resulting in cell survival. However, in cells in which HR is defective, DSBs can be repaired by a more error-prone NHEJ pathway, leading to cell death. RNF144A promotes PARP1 ubiquitination and degradation, resulting in the absence of the drug target for PARP inhibitor olaparib and therefore decreased cellular sensitivity to olaparib. In addition, RNF144A could decrease PARP inhibitor sensitivity through promoting the proteasomal degradation of DNA-PK, which is a major contributor to the cytotoxicity observed in HR-deficient cells treated with PARP inhibitors. BER, base excision repair; DNA-PK, DNA-dependent protein kinase; DSB, double-strand break; HR, homologous recombination; NHEJ, non-homologous end-joining; PARP1, Poly (ADP-ribose) polymerase 1.

As RNF144A affects PARP1 protein abundance (Figure 4), we further demonstrated that the expression levels of RNF144A are associated with cellular sensitivity to olaparib in MDA-MB-231 cells *in vitro* and xenograft mouse tumor models. Although MDA-MB-231 cells express wild-type BRCA1 [48], treatment of pCDH-expressing MDA-MB-231 cells with olaparib suppressed cell growth *in vitro* and *in vivo* (Figure 5). MDA-MB-231 cells may harbor mutations in genes encoding other DNA repair and checkpoint proteins (for example p53) that could render them sensitive to PARP inhibitors. In addition, we found that knockdown of endogenous RNF144A enhances, whereas induced expression of RNF144A decreases cellular sensitivity to olaparib (Figure 5). One possibility for this observation is that overexpression of RNF144A promotes PARP1 degradation, resulting in the absence of the drug target PARP1 for PARP inhibitors and therefore decreased cellular sensitivity to olaparib (Figure 6). In addition, a recent study showed that RNF144A promotes the proteasomal degradation of DNA-PK [36], which is a major contributor to the cytotoxicity observed in HR-deficient cells treated with PARP inhibitors [49]. Thus, we can not rule out the possibility that induced expression of RNF144A decreases cellular responsiveness to PARP inhibitors through downregulation of DNA-PK (Figure 6). In support of our findings presented here, a recent study using complementary genetic screens identified the E3 ubiquitin ligase CBLC as a modifier of PARP inhibitor sensitivity [50]. These emerging evidence highlights that deregulation of the ubiquitin machinery has the potential to influence the response of tumor cells to PARP inhibitors [50].

In summary, we report that RBR-type E3 ubiquitin ligase RNF144A is a novel binding partner of PARP1 and promotes its ubiquitination and proteasomal degradation. In addition, the expression levels of RNF144A in breast cancer cells are associated with breast cancer cellular sensitivity to PARP inhibitor olaparib. These findings may aid in elucidating the mechanistic role for PARP1 in breast cancer pathogenesis and progression and in guiding the selection of optimal patients who are suitable for PARP inhibition therapy.

## MARTIALS AND METHODS

### Cell culture and chemicals

The human breast cancer cell lines MCF-7, T47D, SK-BR-3, BT474, and MDA-MB-231, human breast epithelial cell line HBL100, and human embryonic kidney 293T (HEK293T) cell line were obtained from the Type Culture Collection of the Chinese Academy of Sciences (Shanghai, China). Two highly metastatic variants derived from parental MDA-MB-231 cell line, LM2-4173 and LM2-4175 [38], were kindly provided by Guohong Hu (Shanghai Institutes for Biological Sciences, Chinese Academy of Sciences, Shanghai, China). The LM2-4173 and LM2-4175 cell lines were the second generation *in vivo* selected lung metastatic populations (LM2) from the parental MDA-MB-231 cell line [38]. These cell lines were maintained in DMEM or RPMI1640 medium (Cellgro, Manassas, VA, USA) containing 10% fetal bovine serum (Gibco, Carlsbad, CA, USA), 100 units/ml of penicillin, and 100 μg/ml of streptomycin (Cellgro). Cell lines were expanded and frozen immediately into numerous aliquots after arrival. The cells revived from the frozen stock were used within 10-15 passages and not exceeding a period of 6 months. PARP inhibitor olaparib was obtained from Selleck Chemicals (Houston, TX, USA) and was dissolved in DMSO for *in vitro* experiments and in sterile distilled water containing 4% DMSO and 30% polyethylene glycol for *in vivo* experiments. All chemicals were obtained from Sigma-Aldrich (St. Louis, MO, USA) unless otherwise stated.

### Expression vectors, plasmid transfection, and lentiviral infection

Myc-DDK-tagged-human RNF144A and PARP1 expression vectors, human RNF144A short hairpin RNA (shRNA) in pGFP-C-shLenti vector, and the corresponding control expression vectors were purchased from Origene (Rockville, MD, USA). Human RNF144A shRNA in pGLVH1/GFP+Puro vector was obtained from Genomeditech (Shanghai, China). To generate HA-PARP1 and GST-RNF144A constructs, PARP1 and RNF144A cDNAs were amplified by PCR and then subcloned into pCDH-CMV-MCS-EF1-Puro (System Biosciences, Mountain View, USA) and pGEX-6P-1 expression vectors (kindly provided by Yanhui Xu at Institutes of Biomedical Sciences, Fudan University, Shanghai, China), respectively. To generate Flag-RNF144A, RNF144A cDNAs were amplified by PCR and then subcloned into pSG5 and pCDH-CMV-MCS-EF1-Puro vectors with N-terminal Flag tag. Flag-RNF144A mutations (C20A/C23A and C198A) were generated by PCR-directed mutagenesis. All constructs were verified by sequencing (HuaGene Biotechnology, Shanghai, China). The detailed information of DNA constructs and the primers used for molecular cloning is provided in Supplementary Tables 2 and 3.

Plasmid transfection was carried out using Teng-fect (Tengyi, Shanghai, China) DNA transfection reagents according to the manufacturer’s protocol. For viral infection experiments, HEK293T cells were transfected with each lentivirus expression vector and packaging plasmid mix using Lipofectamine 2000 (Invitrogen, Waltham, MA, USA) or Teng-fect DNA transfection reagents. Media with progeny virus was collected after 48 h of transfection, filtered with 0.45-μm filters (Millipore, Billerica, MA, USA), and either freshly used to infect target cells or stored at -80°C in small aliquots. To generate stable cell lines, cells were infected with lentiviral supernatants diluted 1:1 with culture medium in the presence of 8 μg/ml of Polybrene. After 24 h of infection, cells were selected with 2 μg/ml puromycin (Cayman, Ann Arbor, MI, USA) for 1 week and then passaged before use.

### Immunoblotting, immunoprecipitation (IP), immunofluorescence (IF), and mass spectrometry

The detailed information for primary antibodies used in this study is provided in Supplementary Table 4. Immunoblotting, IP, and liquid chromatography-tandem mass spectrometry (LC-MS/MS) were performed as described previously [51, 52]. For immunoblotting, total cellular lysates were harvested in the modified radioimmunoprecipitation assay (RIPA) lysis buffer (50 mM Tris-HCl, pH 7.4, 150 mM NaCl, 1% NP-40, 0.25% sodium deoxycholate, and 1 mM EDTA) containing 1× protease inhibitor cocktail (Roche, Shanghai, China) and 1×phosphatase inhibitor cocktail (Bimake, Houston, TX, USA). Proteins were isolated on SDS-PAGE gels and transferred to PVDF membrane (Millipore). Immunoblotting was performed by using the indicated antibodies. For IP analysis, cells were lyzed in NP-40 lysis buffer (50 mM Tris-HCl, pH8, 150 mM NaCl, 0.5% NP-40, 10% glycerol, 2 mM MgCl_2_, and 1 mM EDTA) and subjected to IP analysis with 1-3 μg antibody overnight at 4 °C on a rotating platform, followed by immunoblotting analysis. For indirect IF staining, cells grown on glass coverslips were fixed for 15 min with 3.7% formaldehyde. The fixed cells were permeabilized using 0.1% Triton X-100 in PBS for 15 min followed by blocking with 10% goat serum and then incubation with primary antibodies. Cells were mounted with DAPI-containing mounting medium (Abcam, Shanghai, China). Images were captured using a Laser Scanning Confocal Microscope (Leica Microsystems, Buffalo Grove, IL, USA). Identification of the RNF144A-interacting proteins was performed by LC-MS/MS as described previously [52]. Data from LC-MS/MS analysis was searched against Swiss-Prot database by SEQUEST. Trans Proteomic Pipeline software (Institute of Systems Biology, Seattle, WA, USA) was used to identify proteins based on the corresponding peptide sequences with ≧95% confidence. A Protein Prophet 3 probability of 0.95 was used for the protein identification results. The false positive rate was less than 1% [52].

### GST pull-down and *in vivo* ubiquitination assays

The GST pull-down and *in vivo* ubiquitination assay were performed as previously described [51]. For GST pull-down assays, total cellular lysates from HBL100 and BT474 cells were incubated with glutathione-sepharose beads (GE Healthcare, Beijing, China) containing either GST-RNF144A or GST at 4 °C for 4 h. The precipitated proteins were separated by SDS-PAGE and detected by immunoblotting with an anti-PARP1 antibody. For the *in vivo* ubiquitination assay, the HEK293T cells were transfected with the indicated expression vectors. After 42 h of transfection, cells were treated with 10 μM MG-132 for 4 h and then subjected to the sequential IP and immunoblotting analysis with the indicated antibodies.

### Quantitative real-time PCR (qPCR)

Total RNA was isolated using Trizol reagent (Invitrogen) and converted to cDNA using PrimeScript RT Master Mix (Takara, Dalian, China). qPCR analyses were performed using FastStart Universal SYBR Green Master (Roche, Shanghai, China). Primer information is described in Supplementary Table 5.

### Cell viability assay and colony formation assay

To assess cell viability, cells were seeded at 1×10^4^ cells per well in a 96-well plate. Cells were allowed to adhere overnight and then treated with DMSO or olaparib at the indicated concentrations for 48 h. Cell viability was determined by Cell Counting Kit-8 (CCK-8) (Dojindo, Shanghai, China). The absorbance was measured at 450 nm. Analysis of cell viability at each drug dose was expressed as a percentage of cells remaining compared with DMSO treatment group. For colony formation assay, cells (800 cells per well) were plated into 12-well plates. After overnight incubation, cells were treated with DMSO or olaparib at the indicated doses for 7 days. The colonies were fixed and stained with 0.5% crystal violet. Colonies consisting of 50 cells or more were counted.

### Tumorigenesis in nude mice and olaparib administration

MDA-MB-231 cells stably expressing pCDH and Flag-RNF144A (1×10^7^) were injected subcutaneously into the mammary fat pads of 6-8 week old female BALB/c nude mice (State Key Laboratory of Oncogenes and Related Genes, Shanghai Jiaotong University, China). When the tumor volume reached to about 200 mm^3^, mice (n=6) were administrated via intraperitoneal injection with olaparib (50 mg/kg) [40] or vehicle alone. Each animal inthe study received one daily drug administration for five consecutive days, followed by 2 days of no treatment. Tumor size was measured using a vernier caliper every 2 days, and tumor volume was calculated by the formula: 0.5 × length × width^2^ [40]. At the end of experiments, animals were killed by cervical dislocation, and tumor samples were collected. All procedures for animal experiments were approved by the Animal Ethics Committee at Fudan University.

### Statistical analysis

All data are presented as the mean ± standard error from at least three independent experiments. The Student’s t test was used to compare two groups of independent samples. P values of less than 0.05 were considered statistically significant.

## SUPPLEMENTARY MATERIALS TABLES





## Abbreviations

BER: base excision repair
DNA-PK: DNA-dependent protein kinase
DSB: double-strand break
HR: homologous recombination
NHEJ: non-homologous end joining
PARP: poly(ADP-ribose) polymerase
RBR: RING-between-RING
RNF144A: ring finger protein 144A
SSB: single-strand break
