# Supplementary material for: RBR-type E3 ubiquitin ligase RNF144A targets PARP1 for ubiquitin-dependent degradation and regulates PARP inhibitor sensitivity in breast cancer cells
Source: Oncotarget. 2017 Oct 10;8(55):94505–18. doi: 10.18632/oncotarget.21784 (PMC5706891; doi:10.18632/oncotarget.21784)
Supplement: Supplementary file 1 [file oncotarget-08-94505-s001.pdf]

RBR-type E3 ubiquitin ligase RNF144A targets PARP1 for ubiquitin-dependent degradation and regulates PARP inhibitor sensitivity in breast cancer cells

SUPPLEMENTARY MATERIALS

Supplementary Table 1: List of 46 proteins that were identified in Flag-RNF144A immunocomplex

See Supplementary File 1

Supplementary Table 2: Information of expression vectors used in this study

Plasmids	Sources	Vectors
shNC	Origene	pGFP-C-shLenti
shRNF144A	Origene	pGFP-C-shLenti
shNC	Genometech	pGLVH1/GFP+Puro
shRNF144A	Genometech	pGLVH1/GFP+Puro
Myc-DDK-RNF144A	Origene	pCMV6-Entry
Myc-DDK-PARP1	Origene	pCMV6-Entry
Flag-RNF144A	Subcloned	pCDH-CMV-MCS-EF1-Puro
Flag-RNF144A C20A/C23A	Subcloned	pCDH-CMV-MCS-EF1-Puro
Flag-RNF144A C198A	Subcloned	pCDH-CMV-MCS-EF1-Puro
GST-RNF144A 1-292	Subcloned	pGEX-6P-1
GST-RNF144A 1-253	Subcloned	pGEX-6P-1
GST-RNF144A 1-178	Subcloned	pGEX-6P-1
GST-RNF144A 173-292	Subcloned	pGEX-6P-1
GST-RNF144A 173-253	Subcloned	pGEX-6P-1

Supplementary Table 3: Primers for molecular cloning

Expression vectors	Sequences
PSG5-FLAG-144A-F	TACCTCTAGAGAATTCAGCGCCGACACCACCACAACAAGGTACCGG
PSG5-FLAG-144A-R	CGGGCGGCCGCTCGAGCTAGGTGGGTAACGGGTCGT
pCHD-FLAG-144A-F	TAGAGCTAGCGAATTGCCACCATGGCAGACTACAAGGACGACGATGAC
pCHD-FLAG-144A-R	AGATCCTTCGCGGCCCTAGGTGGGTAACGGGTCGT
BamHI-RNF144A-1F	ATTAGGATCCATGACCACAGCAAGGTAC
BamHI-RNF144A-173F	ATTAGGATCCTTCAAAATGGAAGAAGAT
XhoI-RNF144A-178R	ATAACTCGAGCTAATCTTCTTCCATTTT
XhoI-RNF144A-253R	ATAACTCGAGCTA AATGCCCACAACCTGTGT
XhoI-RNF144A-292R	ATAACTCGAGCTAGGTGGGTAACGGGTCG
RNF144A-C20A/C23A F	GCCCTCGACCCGCTGGTGTCTGCCAAGCTCGCTCTTGGGGAG
RNF144A-C20A/C23A R	GTCATCTGCTCCACTGGGTACTCCCCAAGAGCGAGCTTGGCAGACACCA
RNF144A-C198A F	GTGCAAAGTCTACATCGAGCGAGACGAAGGCGCCGCGCAGATGATGTGCAA
RNF144A-C198A R	GCGTGCTTGCAGTTCTTGCACATGCTCTGCGCGCAGCCTTCGTCTCGCTCGAT
HA-PARP1 F	AACCTCTAGAATGGCGGAGTCTTCGGATAAG
HA-PARP1 R	AACCGCGGCTTAAGCGTAGTCTGGGACGTCGTATGGGTACCACAGGGAGGTCTTAAAATTG
V5-Ubiquitin F1	CCTAACCCTCTCCTCGGTCTCGATTCTACGATGCAGATTTTCGTGAAGAACCTTAC
V5-Ubiquitin F2	ACCTCCATAGAAGATTCTAGAGCCACCATGGGTAAGCCTATCCCTATCCCTAACCCTAACCCTCTCCTC
V5-Ubiquitin R	GATCCATTTAAATTCGAATTCTTAACCACCACGAAGTCTCAACACCAAATG

Supplementary Table 4: Information for primary antibodies used in this study

Antibodies	Vendors	Cat#	WB	IP	IF
BRCA1	CST	14823	+		
HA	CST	7392	+	+	+
V5	CST	13202	+		
PARP1	CST	9532	+	+	
RNF144A	LifeSpan	LS-C162648	+		
RNF144A	LifeSpan	LS-C353197			+
Ubiquitin	Millipore	04-263	+		
PARP1	Santa Cruz	sc-8007			+
β-actin	Sigma	A1978	+		
Flag	Sigma	F3165	+	+	+
Vinculin	Sigma	V9131	+		

Supplementary Table 5: Primers for qPCR analysis

Genes	Primers	Sequences
RNF144A	Forward	CCACCTACAGGAGAACGAG
	Reverse	TCCGACAGGGATCAAACA
PARP1	Forward	CTGGGGAGTCGGCGATCTT
	Reverse	GGTTACCCACTCCTTCCGGT
GAPDH	Forward	CGAGATCCCTCCAAAATCAA
	Reverse	TTCACACCCATGACGAACAT
