# Supplementary material for: RBR-type E3 ubiquitin ligase RNF144A targets PARP1 for ubiquitin-dependent degradation and regulates PARP inhibitor sensitivity in breast cancer cells
Source: Oncotarget. 2017 Oct 10;8(55):94505–18. doi: 10.18632/oncotarget.21784 (PMC5706891; doi:10.18632/oncotarget.21784)
Supplement: Supplementary file 2 [file oncotarget-08-94505-s002.docx]

**Supplementary Table 1: List of 46 proteins that were identified in Flag-RNF144A immunocomplex**

| GNA13_HUMAN | Guanine nucleotide-binding protein subunit alpha-13 OS=Homo sapiens GN=GNA13 |
| --- | --- |
| SVIL_HUMAN | Supervillin OS=Homo sapiens GN=SVIL PE=1 SV=2 |
| SFPQ_HUMAN | Splicing factor, proline- and glutamine-rich OS=Homo sapiens GN=SFPQ |
| DHRS2_HUMAN | Dehydrogenase/reductase SDR family member 2, mitochondrial OS=Homo sapiens GN=DHRS2 |
| PP1B_HUMAN | Serine/threonine-protein phosphatase PP1-beta catalytic subunit OS=Homo sapiens GN=PPP1CB |
| SCRIB_HUMAN | Protein scribble homolog OS=Homo sapiens GN=SCRIB |
| E41L3_HUMAN | Band 4.1-like protein 3 OS=Homo sapiens GN=EPB41L3 |
| PLCD3_HUMAN | 1-phosphatidylinositol 4,5-bisphosphate phosphodiesterase delta-3 OS=Homo sapiens GN=PLCD3 |
| PARP1_HUMAN | Poly [ADP-ribose] polymerase 1 OS=Homo sapiens GN=PARP1 |
| CD109_HUMAN | CD109 antigen OS=Homo sapiens GN=CD109 |
| MYL6B_HUMAN | Myosin light chain 6B OS=Homo sapiens GN=MYL6B |
| RS4X_HUMAN | 40S ribosomal protein S4, X isoform OS=Homo sapiens GN=RPS4X |
| LAP2A_HUMAN | Lamina-associated polypeptide 2, isoform alpha OS=Homo sapiens GN=TMPO |
| MY18A_HUMAN | Unconventional myosin-XVIIIa OS=Homo sapiens GN=MYO18A |
| TPM4_HUMAN | Tropomyosin alpha-4 chain OS=Homo sapiens GN=TPM4 PE=1 SV=3 |
| CSKP_HUMAN | Peripheral plasma membrane protein CASK OS=Homo sapiens GN=CASK |
| DDX10_HUMAN | Probable ATP-dependent RNA helicase DDX10 OS=Homo sapiens GN=DDX10 |
| EF2_HUMAN | Elongation factor 2 OS=Homo sapiens GN=EEF2 |
| CYTSB_HUMAN | Cytospin-B OS=Homo sapiens GN=SPECC1 |
| DLG1_HUMAN | Disks large homolog 1 OS=Homo sapiens GN=DLG1 |
| P85A_HUMAN | Phosphatidylinositol 3-kinase regulatory subunit alpha OS=Homo sapiens GN=PIK3R1 |
| RL17_HUMAN | 60S ribosomal protein L17 OS=Homo sapiens GN=RPL17 |
| RS9_HUMAN | 40S ribosomal protein S9 OS=Homo sapiens GN=RPS9 |
| EIF3F_HUMAN | Eukaryotic translation initiation factor 3 subunit F OS=Homo sapiens GN=EIF3F |
| RL10A_HUMAN | 60S ribosomal protein L10a OS=Homo sapiens GN=RPL10A |
| RAP2C_HUMAN | Ras-related protein Rap-2c OS=Homo sapiens GN=RAP2C |
| CTND1_HUMAN | Catenin delta-1 OS=Homo sapiens GN=CTNND1 |
| GCN1L_HUMAN | Translational activator GCN1 OS=Homo sapiens GN=GCN1L1 |
| RAB35_HUMAN | Ras-related protein Rab-35 OS=Homo sapiens GN=RAB35 |
| P85B_HUMAN | Phosphatidylinositol 3-kinase regulatory subunit beta OS=Homo sapiens GN=PIK3R2 |
| CYFP1_HUMAN | Cytoplasmic FMR1-interacting protein 1 OS=Homo sapiens GN=CYFIP1 |
| ADDA_HUMAN | Alpha-adducin OS=Homo sapiens GN=ADD1 |
| CSK_HUMAN | Tyrosine-protein kinase CSK OS=Homo sapiens GN=CSK |
| PALM_HUMAN | Paralemmin-1 OS=Homo sapiens GN=PALM |
| CALL5_HUMAN | Calmodulin-like protein 5 OS=Homo sapiens GN=CALML5 |
| ENPL_HUMAN | Endoplasmin OS=Homo sapiens GN=HSP90B1 |
| EPHA2_HUMAN | Ephrin type-A receptor 2 OS=Homo sapiens GN=EPHA2 |
| SYLC_HUMAN | Leucine--tRNA ligase, cytoplasmic OS=Homo sapiens GN=LARS |
| NFM_HUMAN | Neurofilament medium polypeptide OS=Homo sapiens GN=NEFM |
| PRDX1_HUMAN | Peroxiredoxin-1 OS=Homo sapiens GN=PRDX1 PE=1 SV=1 |
| TF3C2_HUMAN | General transcription factor 3C polypeptide 2 OS=Homo sapiens GN=GTF3C2 |
| MYO5C_HUMAN | Unconventional myosin-Vc OS=Homo sapiens GN=MYO5C |
| PSB5_HUMAN | Proteasome subunit beta type-5 OS=Homo sapiens GN=PSMB5 |
| CAND1_HUMAN | Cullin-associated NEDD8-dissociated protein 1 OS=Homo sapiens GN=CAND1 |
| SYEP_HUMAN | Bifunctional glutamate/proline--tRNA ligase OS=Homo sapiens GN=EPRS |
| XPO2_HUMAN | Exportin-2 OS=Homo sapiens GN=CSE1L |
